# Unsupervised Domain Adaptation for Facial Expression Recognition Using Generative Adversarial Networks

**DOI:** 10.1155/2018/7208794

**Published:** 2018-07-09

**Authors:** Xiaoqing Wang, Xiangjun Wang, Yubo Ni

**Affiliations:** ^1^State Key Laboratory of Precision Measuring Technology and Instruments, Tianjin University, 300072, China; ^2^Key Laboratory of MOEMS of the Ministry of Education, Tianjin University, 300072, China

## Abstract

In the facial expression recognition task, a good-performing convolutional neural network (CNN) model trained on one dataset (source dataset) usually performs poorly on another dataset (target dataset). This is because the feature distribution of the same emotion varies in different datasets. To improve the cross-dataset accuracy of the CNN model, we introduce an unsupervised domain adaptation method, which is especially suitable for unlabelled small target dataset. In order to solve the problem of lack of samples from the target dataset, we train a generative adversarial network (GAN) on the target dataset and use the GAN generated samples to fine-tune the model pretrained on the source dataset. In the process of fine-tuning, we give the unlabelled GAN generated samples distributed pseudolabels dynamically according to the current prediction probabilities. Our method can be easily applied to any existing convolutional neural networks (CNN). We demonstrate the effectiveness of our method on four facial expression recognition datasets with two CNN structures and obtain inspiring results.

## 1. Introduction

Facial expressions recognition (FER) has a wide spectrum of application potentials in human-computer interaction, cognitive psychology, computational neuroscience, and medical healthcare. In recent years, convolutional neural networks (CNN) have achieved many exciting results in artificial intelligent and pattern recognition and have been successfully used in facial expression recognition [1]. Jaiswal et al. [2] present a novel approach to facial action unit detection using a combination of Convolutional and Bidirectional Long Short-Term Memory Neural Networks (CNN-BLSTM), which jointly learns shape, appearance, and dynamics in a deep learning manner. You et al. [3] introduce a new data set, which contains more than 3 million weakly labelled images of different emotions. Esser et al. [4] develop a model for efficient neuromorphic computing using the Deep CNN technique. H-W.Ng et al. [5] develop a cascading fine-tuning approach for emotion recognition. Neagoe et al. [6] propose a model for subject independent emotion recognition from facial expressions using combined CNN and DBN. However, these CNN models are often trained and tested on the same dataset, whereas the cross-dataset performance is less concerned. Although the basic emotions defined by Ekman and Friesen [7], anger, disgust, fear, happy, sadness, and surprise, are believed to be universal, the way of expressing these emotions can be quite diverse across different cultures, ages, and genders [8]. As a result, a well-trained CNN model, having high recognition accuracy on the training dataset, usually performs poorly on other datasets. In order to make the facial expression recognition system more practical, it is necessary to improve the generalization ability of the recognition model.

In this paper, we aim at improving the cross-dataset accuracy of a CNN model on facial expression recognition. One way to solve this problem is to rebuild models from scratch using large-scale newly collected samples. Large amounts of training samples, such as the dataset ImageNet [9] containing over 15 million images, can reduce the overfitting problem and help to train a reliable model. However, for facial expression recognition, it is expensive and sometimes even impossible to get enough labelled training data. Therefore, we proposed an unsupervised domain adaptation method, which is especially suitable for unlabelled small target datasets. Domain adaptation aims at learning knowledge from one dataset (source dataset) and transferring the knowledge to a related but not identical dataset (target dataset). Recent progress involves deep neural networks into the domain adaptation. Long et al. [10] propose a Deep Adaptation Network (DAN) architecture, which generalizes deep convolutional neural network to the domain adaptation scenario. Ganin et al. [11] introduce an unsupervised domain adaptation in deep architectures that can be trained on large amount of labelled data from the source domain and large amount of unlabelled data from the target domain. Tzeng et al. [12] propose a new CNN architecture to exploit unlabelled and sparsely labelled target domain data. Our method also uses CNN as the basic structure. But unlike [11], which needs large-scale unlabelled data from the target domain, our method works well with small-size unlabelled target dataset by using GAN generated samples.

The generative adversarial networks (GAN) have two subnetworks: a generator and a discriminator. The discriminator decides whether a sample is generated or real, while the generator produces samples to cheat the discriminator. The GAN is first proposed by Goodfellow et al. [18]. DCGAN [19] combines GAN with CNN and provides techniques to improve the training stability. InfoGAN [20] learns interpretable representations by introducing latent codes. WGAN [21] introduces Wasserstein distant to replace the KL divergence, which solves the model collapse problem in GAN and produces GAN samples with higher diversity. In this work, we use a GAN model similar to the one used in the WGAN to generate unlabelled samples from the target data and these samples will help the baseline CNN to gain a better knowledge of the target distribution. Actually, we are not the first to introduce GAN generated samples into CNN training. Odena [22] treats GAN samples as a new class during a semisupervised training. Zheng et al. [23] also focus on semisupervised training and assign a uniform label distribution over all the existing classes to GAN samples. Unlike these methods, we give GAN samples distributed pseudolabels, which have different weights with different classes and we change the weights dynamically according to the CNN prediction probability vector during training.

In this paper, the dataset that is used to train the baseline CNN is referred to as the source dataset, and the dataset being tested on for cross-dataset performance is referred to as target dataset. Our method uses samples generated by a generative adversarial network (GAN) to make up for the shortage of samples in the target dataset. More specifically, we apply our method on two widely used CNN structures, Alexnet [24] and VGG11 [25], and we train a CNN model as baseline using the source dataset, but our method is not limited with these two models and can be easily applied to other CNN models as well. Then we train a GAN using the target dataset to generate GAN samples. These unlabelled newly generated samples are combined with the source dataset to fine-tune the CNN baseline model to help the model to get a better recognition accuracy on the target domain. During fine-tuning, a distributed pseudolabel (DPL) is given to the newly generated sample according to its current prediction probabilities. We evaluate our method on the cross-dataset facial expression recognition task with four datasets. Experiments have shown that our method obtains state-of-the-art results on cross-dataset FER. The main contributions of this paper are as follows:

- Introducing an unsupervised domain adaptation method using GAN generated samples.
- Proposing a distributed pseudolabel method for samples generated by GAN.
- Improving the cross-dataset accuracy of baseline CNN in facial expression recognition using the proposed method.

## 2. Proposed Method

### 2.1. Overall Architecture

**Figure 1:**
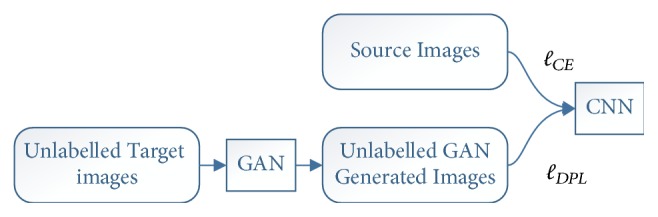
Overall training structure of the domain adaptation, *ℓ*_*CE*_ is the cross-entropy loss used for the images in the source dataset and *ℓ*_*DPL*_ is the distributed pseudolabel loss used for the unlabelled GAN images.

The overall architecture of our unsupervised domain adaptation is shown in Figure 1. We first train a facial expression recognition CNN with the source images. After training a CNN with the source dataset, we want to improve its cross-dataset performance without the ground truth label information of the target dataset. To this end, we must deal with the limited number of samples in the target dataset. GAN provides a solution for us. By training a GAN with the target dataset, we can generate more images that follow the same distribution as the target dataset. Then the CNN model can be fine-tuned with the combination of these generated images and the source dataset. Here we include the source dataset during fine-tuning because we find that the experiment results are better compared with those of fine-tuning with only the generated samples. The generated images, however, cannot be directly used to train a CNN because they are unlabelled. Inspired by Szegedy et al.'s [26] label smoothing regularization (LSR) used for supervised learning, we propose a distributed pseudolabel method (DPL) for our unlabelled GAN generated samples.

### 2.2. Distributed Pseudolabel

In a supervised training task, it is classic to use cross-entropy loss during training. Let *k* = {1,2,…, *N*} be the predefined classes, where *N* is the number of classes. The cross-entropy loss function is as follows:

(1) $$\begin{array}{c} l=-\overset{N}{\underset{k=1}{\sum }}q\left(\;k\;\right)\log\frac{\exp\left(x_{k}\right)}{\sum_{i=1}^{N}\exp\left(x_{i}\right)}=-\overset{N}{\underset{k=1}{\sum }}q\left(\;k\;\right)\log\left(\;p\left(\;k\;\right)\;\right) \end{array}$$

where *x*_*i*_, *i* ∈ {1,2,…, *N*} is the output of the CNN's last fully connected layer. *p*(*k*) is the prediction probability of the input image belonging to class *k*, which is the normalized value of *x*_*k*_. *q*(*k*) is the label distribution of the input image.

Let *k*_*label*_ be the ground truth class; the one-hot label style *q*(*k*) is defined as follows:

(2) $$\begin{array}{c} q\left(\;k\;\right)=\left\{\begin{array}{cc} 1 & k=k_{label}\\ 0 & k\neq k_{label} \end{array}\right. \end{array}$$

With (2), the loss function in (1) becomes

(3) $$\begin{array}{c} l_{CE}=-\log\left(\;p\left(\;k_{label}\;\right)\;\right) \end{array}$$

**Figure 2:**
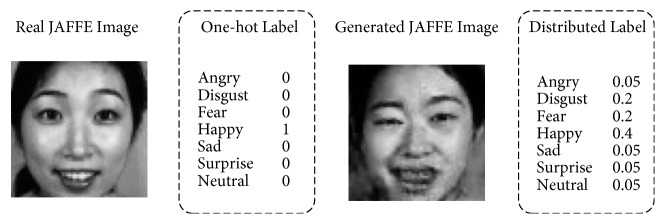
The one-hot label of real JAFFE Image and the distributed label of a generated JAFFE image used in our DPL method.

Unlike supervised training, our GAN generated images has no ground truth class. Therefore, before using them to fine-tune the CNN model, we need to give them proper labels. We proposed our method based on an observation that each of the generated samples is a mixture of different people and different emotions from the target dataset, for example, a mouth from a happy person A, an eye from a sad person B, and another eye from a fear person C. This is because these GAN generated images randomly contain features learned from the target dataset, on which the GAN was trained. In the meantime, a generated image will be more similar to a certain emotion than the others. The generated image in Figure 2, for example, is more similar to an original happy face than other emotions. As a result, instead of giving them one-hot labels, we use distributed pseudolabels with different weights to different classes. This idea is inspired by the label smoothing method [26], in which *q*(*k*) is defined as follows:

(4) $$\begin{array}{c} q\left(\;k\;\right)=\left\{\begin{array}{cc} 1-\varepsilon +\frac{\varepsilon }{N} & k=k_{label}\\ \frac{\varepsilon }{N} & k\neq k_{label} \end{array}\right. \end{array}$$

where *ε* is a hyperparameter set to 0.1.

By applying (4) to (1), the cross-entropy loss function evolves to

(5) $$\begin{array}{c} l_{LSR}=-\left(\;1-\varepsilon \;\right)\log\left(\;p\left(\;k_{label}\;\right)\;\right)-\frac{\varepsilon }{N}\overset{N}{\underset{k=1}{\sum }}\log\left(\;p\left(\;k\;\right)\;\right) \end{array}$$

Label smoothing assigns small values to the non-ground truth classes instead of 0. This strategy discourages the network to be tuned toward the ground truth class and thus reduces the chances of overfitting. As for the distributed label for our unlabelled GAN samples, we feed a generated sample into the CNN model and use the prediction probability vector to help us decide which class gets a higher weight. Since the baseline CNN model is trained on the source dataset, its prediction on our generated samples, which are generated from the target dataset, cannot be highly reliable. Therefore, instead of giving higher weight to only one class, we give higher weights to three classes which have the top 3 maximum prediction probabilities. We refer to this method as distributed pseudolabel method (DPL).

Let *k*_*top*1_, *k*_*top*2_, and *k*_*top*3_ be the classes that have the top 3 maximum prediction probabilities after a generated image passes through the CNN. Our distributed pseudolabel for this generated image is

(6) $$\begin{array}{c} q\left(\;k\;\right)=\left\{\begin{array}{cc} \lambda_{1} & k=k_{top1}\\ \lambda_{2} & k=k_{top2}\\ \lambda_{3} & k=k_{top3}\\ \frac{1-\lambda_{1}-\lambda_{2}-\lambda_{3}}{N-3} & otherwise \end{array}\right. \end{array}$$

where *λ*_1_, *λ*_2_, and *λ*_3_ are hyperparameters set to 0.4, 0.2, and 0.2 in our experiments.

During training, each time when a GAN generated image passes through the CNN, we assign a new distributed pseudolabel to it according to the current prediction, so the label of the generated image can change dynamically. With DPL, the entropy loss function for GAN generated images changes to (7), whereas the images from the source dataset still use the one-hot labels during training and their loss function is (3). Since the GAN images have no ground truth labels and their prediction results from CNN based on the source dataset cannot be highly accurate, DPL gives them the top 3 most likely classes they belong to and encourages the network to be tuned toward these classes with major consideration and thus help the network to gain a better recognition accuracy on the target dataset. The fine-tuning process using DPL is illustrated in Algorithm 1.

(7) lDPL=−λ1log⁡pktop1−λ2log⁡pktop2−λ3log⁡pktop3−1−λ1−λ2−λ3N−3∑k=1k≠ktop1,ktop2,ktop3Nlog⁡pk

## 3. Implementation Details

### 3.1. Datasets

**Figure 3:**
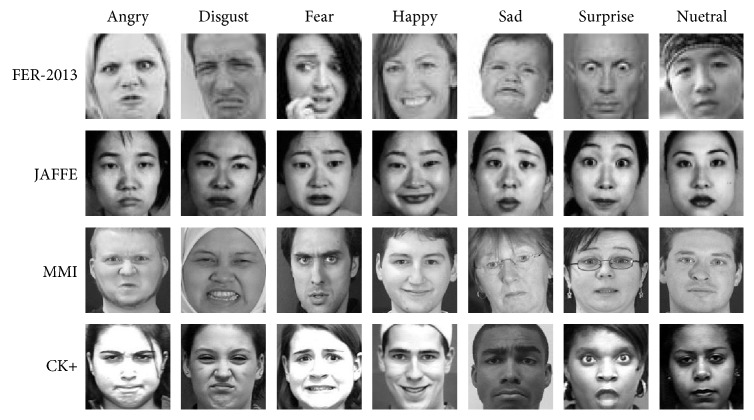
Sample images from four datasets.

We use four FER datasets and seven emotions in our experiments.  Figure 3 shows sample images from these datasets.

**FER-2013** is a large-scale FER dataset used in the ICML 2013 workshop's facial expression recognition challenge [27]. The dataset has seven expressions including anger, disgust, fear, happy, sad, surprise, and neutral, and it comprises three parts: the training data (FER-TRA), which consists of 28709 images, the Public test data (FER-PUB), which consists of 3859 images, and the private test data (FER-PRI), which also consists of 3859 images. The images of FER-2013 were collected from the Internet and the faces greatly vary in age, pose and occlusion, thus resulting the accuracy of human recognition is only approximately 65 ± 5% [28]. As a powerful machine learning tools, the CNN can now surpass human beings on the FER-2013 task, and the state-of-the-art accuracy on FER-2013 is 75.42% by combining CNN extracted features and handcrafted features for training [29]. In this paper, we apply our method to two less fancy yet more commonly used CNN architectures, namely, Alexnet [24] and VGG11 [25]. When we use the FER-TRA for training and use the FER-PRI for testing, the recognition accuracy we get is 66.37% and 65.03% respectively.

**JAFFE** dataset consists of 213 facial expression images from 10 Japanese females [30, 31]. They posed seven basic expressions (anger, disgust, fear, happy, sad, surprise, plus neutral expression). We use all of the images in JAFFE, either as source dataset or as target dataset in different experiments.

**CK+** dataset consists of 593 sequences from 123 subjects, among which 327 sequences have emotion labels [32]. The dataset contains seven expressions including anger, disgust, fear, happy, sad, surprise, and contempt. We only chose the peak frame from the sequences labelled with the first six expressions. In addition, we chose the first frame from some of the sequences as neutral samples. In total we use 363 images from CK+. The CK+ dataset is used as source dataset as well as target dataset.

**MMI **dataset consists of over 2900 videos and high resolution still images of 75 subjects, in which 235 videos have emotional labels [33, 34]. We chose the peak frame of each video with the six basic emotions and the first frame as neutral emotion. In total we use 242 images from MMI. The MMI dataset was only used as target dataset in our experiments.

### 3.2. Network Structure

The Alexnet [24] and VGG11 [25] architectures are used as the CNN for expression recognition in our experiment, and, with each model, we modify the last fully connected layer to have 7 neurons to predict the seven emotion classes. We detect and crop the faces out of JAFFE, CK+, and MMI but we do not crop the FER2013 because the original images are too small (48 × 48). All the images are resized into 224 × 224 before training. We train the CNNs on the source dataset and test them on the target dataset. The cross-dataset accuracy of these models is used as our experiment baseline, which are shown in Table 1. And these CNN models are used as the pretrained models for fine-tuning in the domain adaptation process. During training, we use stochastic gradient descent with the learning rate set to 0.00001 and the momentum set to 0.9. We train the Alexnet for 50 epochs and the VGG11 for 100 epochs. The CNN models are all trained on Pytorch.

**Figure 4:**
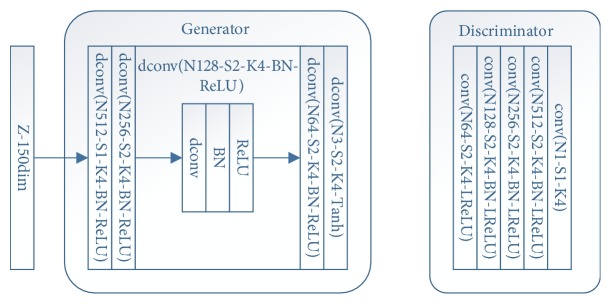
Network structure of GAN. The convolutional layer is denoted as conv and the transposed convolutional layer is denoted as dconv. N stands for neurons (channels), S stands for stride, and K stands for kernel size. LReLU means leaky ReLU nonlinearity and BN means batch normalization.

**Figure 5:**
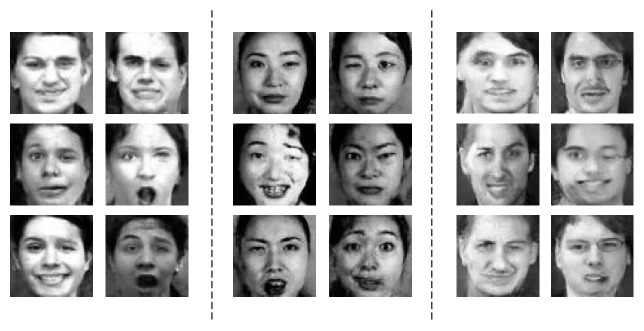
From left to right, GAN generated images from CK+, JAFFE, and MMI.

We resize the face-cropped images of CK+, JAFFE, and MMI to 64 × 64 for GAN training. The GAN structure is showed in Figure 4. Following [21], we use Wasserstein distance to calculate the loss during training. The input vector z is set to 150-dim, and for each GAN model we train 5000 epochs. The GAN models are also trained on Pytorch. Figure 4 shows the GAN structure used in our experiment.  Figure 5 shows some of the samples of our GAN generated images.

In the domain adaptation training process, we use 2k GAN images in each experiment. The weights of the top 3 classes (*λ*_1_, *λ*_2_, and *λ*_3_) in DPL are set as 0.4, 0.2, and 0.2, respectively.

## 4. Experiment Results and Discussion

### 4.1. Experiment Results

We conduct a series of experiments over different datasets. During training, the source dataset and its label information is used to train the CNN, whereas the target dataset is only used to generated GAN samples without the label information. The label information of the target dataset is only used for testing. First, the relatively large dataset, FER-2013, is used as source dataset. When using Alexnet as the CNN structure, we take JAFFE, MMI, and CK+ as target dataset and obtain 3.76%, 3.72%, and 4.41% recognition accuracy improvement on the target dataset, respectively. We also train on VGG11 with FER-2013 as the source dataset and JAFFE as the target dataset to examine our method on a different CNN structure, and the recognition accuracy increases by 15.02%. Then we use smaller dataset as source dataset to further test our method. When we use CK+ as the source dataset, we get 4.70% improvement of recognition accuracy on JAFFE and 4.68% improvement when using JAFFE as source dataset and CK+ as target dataset. The experiment results have shown that our method can improve the CNN model's recognition accuracy on the target dataset with different datasets as well as different CNN structures.

### 4.2. Comparison with Other Published Method

We compare our experiment results with other published cross-dataset recognition accuracy results in Table 2, and Table 2 shows that our method outperforms most of the published results. When JAFFE is the target dataset, Zhu et al. achieve higher accuracy (61.97%) than our method (59.62%), but they use part of the JAFFE datasets with ground truth labels for transfer learning, whereas our method requires no ground truth labels from the target data at all.

### 4.3. Comparison of Confusion Matrix

We compare the confusion matrix of our result with the baseline CNN trained only with the source dataset to see the recognition accuracy changes of each class of the emotions. In Tables 3 and 4, the source dataset is FER-2013, the target dataset is JAFFE, and the CNN structure is VGG11.  Table 3 shows that the baseline CNN trained on FER2013 performs poorly on JAFFE and has a tendency of classifying the JAFFE images as Neutral. More specifically, it misclassifies all the Anger images as Neutral and has low classification accuracy on Disgust and Sad, only 13.79% and 16.13% respectively, whereas 55.5% and 80.65% of these two emotions are misclassified as Neutral.  Table 4 shows that, after applying our method, the Angry, Disgust, and Sad accuracy improves to 23.33%, 58.62%, and 32.26%, respectively, which certifies that our method can effectively improve the baseline CNN's understanding of the target domain.

### 4.4. Comparison with Two Other GAN-Based Domain Adaptation Methods

We compare DPL with two alternative methods using GAN generated images, the pseudolabel [35], and the LSRO [23].

- Pseudolabel takes the class which has the highest predicted probability as the unlabelled image's one-hot pseudolabel and updates the pseudolabel each time when the unlabelled image is fed into the network.
- LSRO is a regularization method used for GAN samples generated from a person re-ID dataset; they presume that the generated samples do not belong to any of the person predefined and should be labelled with a uniform distribution *q*(*k*) = 1/*k* over all the classes.

This experiment is conducted using both two CNN architectures, the Alexnet and the VGG11, with 2k GAN images. The source dataset is FER-2013 and the target dataset is JAFFE.  Table 5 shows that pseudolabel does not work well on the FER task and even decreases the cross-dataset accuracy on VGG11. LSRO does improve the model's accuracy on the target dataset, but our method has the best results on both networks.

### 4.5. Comparison with Real Images

In previous experiments, we only fine-tune the CNN with GAN generated samples; now we want to investigate how our method performs with real images from the target dataset. We use FER-2013 as the source dataset and train a CNN with it. And we treat the JAFFE as unlabelled target images to fine-tune the CNN with DPL. Since the JAFFE dataset has only 213 images, we fine-tune a CNN with 213 generated images for comparison.  Table 6 shows that our method works on unlabelled real images as well, and the real-world images actually achieve better cross-dataset accuracy on VGG11 (56.34%) compared with the result trained with the same amount of GAN generated samples (55.87%). But limited by the total number of images, the results with real 213 images are far below our results with 2k generated samples. Then we combine 213 real images and 2k GAN generated images to fine-tune the CNN. This strategy slightly outperforms our best results with 2k generated images by a margin of 0.94% and 0.47% on Alexnet and VGG11, respectively. These experiment results indicate that our DPL method not only can be used on domain adaptations with GAN generated samples but also can be used on unsupervised learning tasks with real-world images.

### 4.6. Weight Parameters for DPL

**Figure 6:**
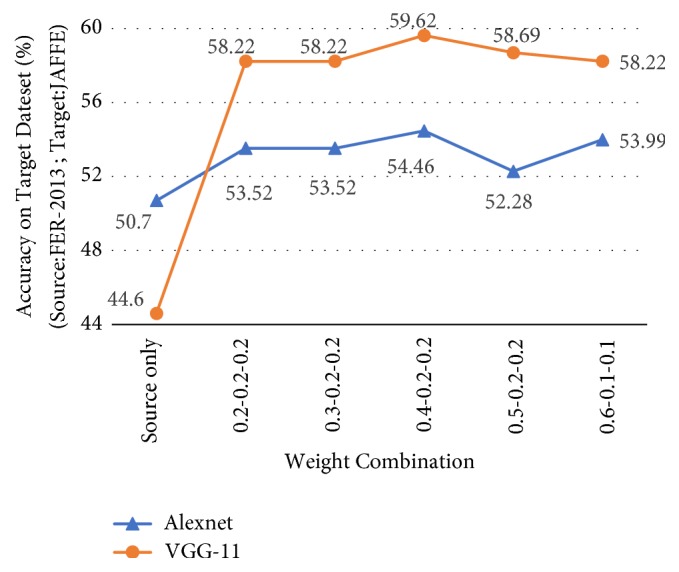
The experiment result with different weight combinations (*λ*_1_-*λ*_2_-*λ*_3_).

The weights of the top 3 classes (*λ*_1_, *λ*_2_, and *λ*_3_) are hyperparameters in DPL.  Figure 6 shows the experiment result with different weight combinations. In this experiment, the source dataset is FER-2013, and the target dataset is JAFFE. During training, we combine 2k GAN images with the source dataset to fine-tune the CNN baseline model. We set the learning rate to 0.000001 and stop fine-tuning at 10 epochs.  Figure 6 shows that the 0.4-0.2-0.2 combination achieves the best result with the target dataset on both models.

### 4.7. The Number of GAN Samples

**Figure 7:**
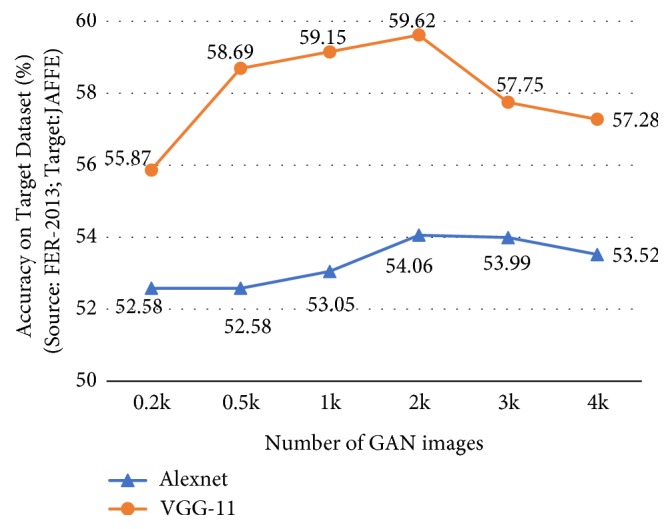
The experiment result using different number of GAN images.

Here we look into the impact of the number of GAN generated images used for DPL on the experiment results. We take FER-2013 as source dataset and JAFFE as target dataset. The 0.4-0.2-0.2 weights combination is used for DPL and the learning rate is set to 0.000001. We stop fine-tuning after 10 epochs. From Figure 7 we can see that, at first, on both VGG11 and Alexnet, the recognition accuracy of the target dataset increases with the number of the generated samples. But after it peaks at 2k images, the accuracy improvement falls again. This is because, when the GAN images are too few, it is inadequate to fine-tune a CNN model toward the target dataset, whereas when the GAN images are too many, the CNN will give too much effort to classify those generated images with pseudolabels. The pseudolabels are not as trustworthy as the ground truth labels and we do not want a CNN to take them too seriously.

## 5. Conclusion

In this paper, we propose an unsupervised domain adaptation method, a method using GAN generated samples to improve the cross-dataset performance of facial expression recognition. When training the CNN with unlabelled GAN generated samples, we introduce a distributed pseudolabel method (DPL). With our method, domain adaptation can be achieved with limited target data without ground truth labels. Experiments have shown that our method outperforms other GAN-based domain adaptation methods and can get state-of-the-art cross-dataset recognition accuracy. When using FER-2013 as the source dataset, we obtain 15.02%, 3.76%, 3.72%, and 4.41% recognition accuracy improvement on the target dataset JAFFE (VGG11), JAFFE (Alexnet), MMI, and CK+, respectively. When using CK+ as the source dataset, we obtain 4.70% improvement of recognition accuracy on JAFFE and 4.68% improvement when using JAFFE as source dataset and CK+ as target dataset. Future work may extend the unsupervised DPL to a semisupervised version since the real-world samples with ground truth label in target dataset might provide better estimation of the target data. Also, it will be intriguing to apply our method to other domain adaptation tasks.

## Figures and Tables

**Algorithm 1:**
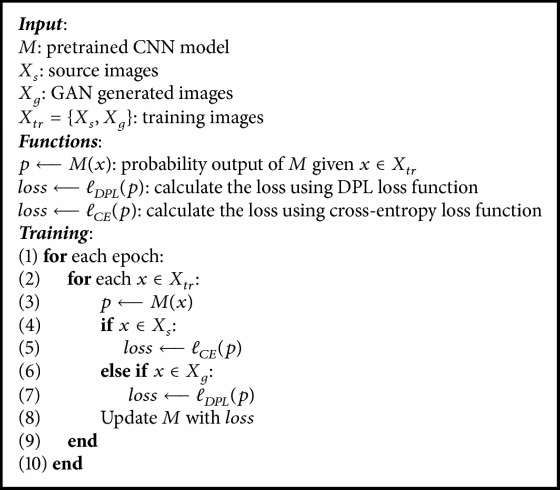
Fine-tuning process using DPL.

**Table 1:** Experiment results of the recognition accuracy on the target dataset.

Model	Source Dataset	Target Dataset	Source Only	Our Result
VGG11	FER-2013	JAFFE	44.60%	59.62%
Alexnet	FER-2013	JAFFE	50.70%	54.46%
Alexnet	FER-2013	MMI	58.14%	61.86%
Alexnet	FER-2013	CK+	71.90%	76.58%
Alexnet	CK+	JAFFE	46.94%	51.64%
Alexnet	JAFFE	CK+	60.33%	65.01%

**Table 2:** Comparison with other published methods.

Method	Source Dataset	Target Dataset	Recognition Accuracy on The Target Dataset
Meguid et al. [13]	Bu-3DFE	JAFFE	41.96%
Wen et al. [14]	FER2013	JAFFE	50.70%
Gu et al. [15]	CK	JAFFE	55.87%
Zhu et al. [16]	FEED	JAFFE	**61.97**%
Our Method	CK+	JAFFE	51.64%
Our Method	FER2013	JAFFE	59.62%

Mayer et al. [17]	CK	MMI	60.30%
Mayer et al. [17]	FEED	MMI	58.90%
Our Method	FER2013	MMI	**61.86**%

Gu et al. [15]	JAFFE	CK+	54.05%
Mayer et al. [17]	FEED	CK+	56.60%
Wen et al. [14]	FER2013	CK+	76.05%
Our Method	JAFFE	CK+	65.01%
Our Method	FER2013	CK+	**76.58**%

**Table 3:** The target dataset recognition accuracy (%) confusion matrix of baseline CNN, FER-2013→JAFFE.

	Angry	Disgust	Fear	Happy	Sad	Surprise	Neutral
Angry	**0.00 **	0.00	0.00	0.00	0.00	0.00	100.00
Disgust	0.00	**13.79**	3.45	0.00	27.59	0.00	55.17
Fear	0.00	0.00	**34.38**	3.13	0.00	9.38	53.13
Happy	0.00	0.00	0.00	**64.52 **	0.00	3.23	32.26
Sad	3.23	0.00	0.00	0.00	**16.13**	0.00	80.65
Surprise	0.00	0.00	0.00	6.67	0.00	**83.33 **	10.00
Neutral	0.00	0.00	0.00	0.00	0.00	0.00	**100.00**

**Table 4:** The target dataset recognition accuracy (%) confusion matrix of our method, FER-2013→JAFFE.

	Angry	Disgust	Fear	Happy	Sad	Surprise	Neutral
Angry	**23.33 **	13.33	0.00	0.00	10.00	0.00	53.33
Disgust	6.90	**58.62 **	3.45	0.00	17.24	0.00	13.79
Fear	9.38	31.25	**50.00 **	0.00	0.00	6.25	3.13
Happy	0.00	0.00	0.00	**96.77 **	0.00	0.00	3.23
Sad	25.81	9.68	16.13	3.23	**32.26**	3.23	9.68
Surprise	0.00	0.00	6.67	13.33	0.00	**80.00 **	0.00
Neutral	6.67	0.00	0.00	6.67	6.67	3.33	**76.67 **

**Table 5:** Comparison with two other methods using GAN generated images, FER-2013→JAFFE.

Method	Recognition Accuracy on Target Dataset	
	Alexnet	VGG11
Baseline	50.70%	44.60%
Pseudo-label	51.17%	42.72%
LSRO	53.99%	57.75%
DPL	**54.46**%	**59.62**%

**Table 6:** Comparison with real images, FER-2013→JAFFE.

Method	Recognition Accuracy on Target Dataset	
	Alexnet	VGG11
Sour-only	50.70%	44.60%
Real-213	51.64%	56.34%
GAN-213	52.11%	55.87%
GAN-2k	54.46%	59.62%
GAN-2k+Real-213	55.40%	60.09%

## Data Availability

The datasets used during the current study are available in the following repository: http://www.kasrl.org/jaffe.htmlhttps://mmifacedb.eu/https://www.kaggle.com/c/challenges-in-representation-learning-facial-expression-recognition-challengehttp://www.pitt.edu/~emotion/ck-spread.htm.
